# Evaluation of Mechanical Properties of Composite Material with a Thermoplastic Matrix Reinforced with Cellulose Acetate Microfibers

**DOI:** 10.3390/polym16182557

**Published:** 2024-09-10

**Authors:** Pedro Rodríguez Sandoval, Andres Felipe Rubiano-Navarrete, Edwin Yesid Gómez-Pachón, Ricardo Vera-Graziano

**Affiliations:** 1 Grupo de Investigación de Materiales y Ensayos—GIMES, SENA—Centro de Materiales y Ensayos, Escuela de Posgrado en Ingeniería, Universidad Pedagógica y Tecnológica de Colombia, UPTC, Tunja 150003, Colombia; pedro.rodriguez01@uptc.edu.co; 2 Grupo de Investigación en Diseño, Innovación y Asistencia Técnica para Materiales Avanzados-DITMAV, Maestría en Metalurgia y Ciencia de los Materiales, Universidad Pedagógica y Tecnológica de Colombia-UPTC, Tunja 150003, Colombia; 3 Grupo de Investigación en Diseño, Innovación y Asistencia Técnica para Materiales Avanzados-DITMAV, Escuela de Diseño Industrial, Universidad Pedagógica y Tecnológica de Colombia-UPTC, Duitama 150461, Colombia; edwin.gomez02@uptc.edu.co; 4 Instituto de Investigaciones en Materiales, Universidad Nacional Autónoma de México, Mexico City 04510, Mexico

**Keywords:** extrusion, low-density polyethylene, microfibers, polymer, reinforcement

## Abstract

Low-density polyethylene (LDPE) has been widely used in various applications due to its flexibility, lightness, and low production cost. However, its massive use in disposable products has raised environmental concerns, prompting the search for more sustainable alternatives. This study aims to investigate the mechanical properties achievable in a composite material utilizing low-density polyethylene (LDPE), potato starch (PS), and cellulose microfibrils (MFCA) at loadings of 0.05%, 0.15%, and 0.30%. Initially, the cellulose acetate microfibrils (MFCA) were produced via an electrospinning process. Subsequently, a dispersive mixture of the aforementioned materials was created through the extrusion and pelletizing process to form pellets. These pellets were then molded by injection molding to produce test specimens in accordance with ASTM D 638, the standard for tensile strength testing. The evaluation of the properties was conducted through mechanical tensile tests (ASTM D638), hardness tests (ASTM D 2240), melt flow index (ASTM D1238), and scanning electron microscopy (SEM). This study determined the influence of cellulose acetate microfibril loadings below 0.3% as reinforcement within a thermoplastic LDPE matrix. It was demonstrated that these microfibrils, due to their length-to-diameter ratio, contribute to an enhancement in the mechanical properties.

## 1. Introduction

Composite materials have become a formidable alternative across various industrial sectors, which is attributable to their high mechanical strength, stiffness, thermal resistance, and hardness, among other properties. These composites are characterized by a blend of materials that achieve emergent properties necessary for specific applications [1,2]. Recently, research has concentrated on developing composite materials endowed with the ability to degrade over time, as a result of interaction with organic matter and microbial agents. The majority of these materials are formulated using a synthetic polymer matrix reinforced with natural fibers or particles [3,4].

The pursuit of the ideal material has led to experiments with reinforcements or fillers that enhance physical properties without compromising their degradability. Consequently, there has been a pivot towards using natural fibers due to their low cost [5,6,7,8], ready availability, and the benefit of utilizing natural waste. Some commonly used fibers for fillers or reinforcements are those derived from coconut, fique, hemp, cocoa, among others. This is a result of the amalgamation of mechanical properties, stiffness, low thermal expansion coefficient, and chemical inertia [9].

The incorporation of microfibrils as reinforcements in materials has significantly contributed to advancements in the creation of materials, as research has focused on micrometric scale reinforcements to ensure better compatibility with synthetic matrices and natural polymers. Various types of micrometric reinforcements exist; however, those of a cellulosic nature have been the most extensively studied and utilized due to cellulose being one of the most abundant structural polymers on earth. Additionally, they exhibit high tensile strength, stiffness, flexibility, and superior mechanical, electrical, thermal, and dynamic properties compared to other commercial fibers [10,11], which render them an ecologically sound reinforcement alternative.

Microfibril acquisition is accomplished through various methods, one of which is electrospinning. This technique involves connecting a polymer solution, contained within a syringe with a metallic needle, to a high-voltage power source where an electrical charge is applied (positive pole), with a metallic collector (negative pole) situated at the other end, typically made of aluminum or copper. This is where the microfibrils are deposited [6].

The electrospinning process begins when voltage is applied at the tip of the needle, creating a droplet in the shape of a cone, which is a consequence of its electrostatic polarization [12]. Once the electric field’s force exceeds the surface tension of the polymer solution, it is propelled toward the collector in the form of a filament. En route to the collector, the solvent evaporates, leading to the formation of a microfibril that is deposited on the collector, subsequently forming a nonwoven membrane [13].

To integrate microcompounds into matrices, a transformation process is necessary to facilitate their incorporation. This has been achieved using various polymer transformation processes such as injection molding, compression, thermoforming, and extrusion, with the latter being the most widely utilized [14,15,16]. Extrusion is a high-temperature, short-time process involving thermal, pressure, and mechanical shearing [17]. This continuous process allows for product quality control by adjusting the temperature and the screw speed [18], making it a versatile transformation process applicable across different industrial sectors.

In this study, an examination of the mechanical properties of a composite material composed of PE/starch reinforced with cellulose microfibers obtained via electrospinning was conducted. The composite material was produced by the extrusion process using a twin-screw extruder for this purpose.

## 2. Materials and Methods

### 2.1. Materials

For the preparation of the composite material, low-density polyethylene (LDPE) from ECOPETROL, Bogotá, Colombia (this low-density polymer is a synthetic polymer derived from petroleum), reference POLIFÉN 641; modified potato starch (PS) from EMSLAND GROUP, potato starch reference; Polyethylene glycol—PG, from CHEMICOL CH S.A.S, reference 6000—batch 200115W001203; and distilled water from CEPROSA, reference distilled, were employed.

The cellulose acetate microfibers (MFCA) were manufactured using the following materials: Cellulose acetate from SIGMA ALDRICH, reference 419028; Acetone from SIGMA ALDRICH, reference 179973-4L; Ethanol from EMSURE, reference 100983-100; and Chloroform from J.T. Baker, reference 918003-4L.

### 2.2. Preparation of Raw Materials

Electrospinning Process

For the preparation of the electrospinning material solution, an exploratory experimental design was conducted that included variations in polymer concentrations and the solvent mixture ratio. As a result of various exploratory tests, a standard concentration of 6.5% cellulose acetate polymer and a solvent ratio of 2:1 Acetone/Chloroform were established.

The laboratory equipment used included digital scales (brands: RADWAG, reference: ac 220/C/2; AVANPRO, reference: ABS), magnetic stirrers (brands: STABLETEMP, reference: COLE-PAEMER; TENAL, reference: TE-0854), 50 mL beakers, graduated cylinders, universal stands, watch glasses, glass jars with plastic lids, and magnets. The cellulose acetate microfibers (MFCA) were obtained by the electrospinning process using a high-voltage power supply (HV350R), injection pump systems Inc NE-1000 (Farmingdale, NY, USA), a copper collector covered with aluminum, a 10 mL Plasticak brand plastic syringe, and a steel needle with a capillary diameter of 0.8 mm developed and constructed by the DITMAV research group of the Universidad Pedagógica y Tecnológica de Colombia UPTC.

For this process, the variables for obtaining the microfibers were established, taking into account previous research, and the process was verified for the homogeneous membrane formation by setting the parameters to a flow rate of 30 mL/h, a distance of 30 cm, and a voltage of 17 KV, as shown in Table 1 [14]. Once the membrane was obtained, a micrograph was taken and the fiber diameter was measured using ImageJ (version 64-bit Java 8) software.

Extrusion/Pelletizing Process

The production of the composite material, composed of a thermoplastic matrix of LDPE/PS reinforced with cellulose acetate microfibers, was conducted at the SENA—Materials and Testing Laboratory.

Subsequently, the measurement of the mass of LDPE, PS, and MFCA commenced, bearing in mind that PS should constitute 15% by weight of the mixture and the proportion of LDPE is directly dependent on the MFCA percentages of 0.05%, 0.15%, and 0.30% of the total mixture weight, resulting in the treatments outlined in Table 2.

The materials were integrated using a SHINI brand vertical-type industrial mixing hopper at a speed of 120 rpm for 10 min, after which they were packaged in bags labeled according to the treatments of the experimental design.

This process began with the setup and programming of the extrusion/pelletizing line in accordance with the treatments listed in Table 3, which details process variables such as temperatures, pressures, speeds, screw rpm, and cycle times.

**Figure 1:**
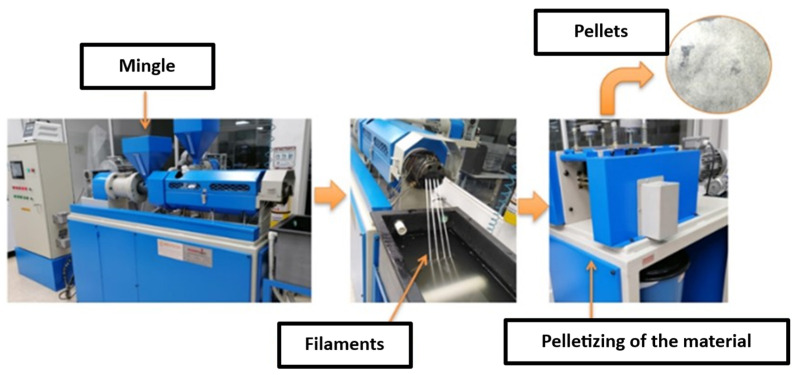
Extrusion/pelletizing process for reinforced material.

A double-screw extruder, series 14198 reference PTL-30, was utilized, which was programmed with a heating curve ranging from 50 to 165 °C across 5 zones and a screw rotation speed of 40 rpm. The pre-heated mixture at 50 °C was added to the machine’s feed hopper and passed through the cylinder, with the screws conveying it to the head where it exits through four dies forming plastic filaments. These filaments then pass through a cooling tank where water under pressure circulates in a closed loop to solidify the material, which is subsequently transported to the pelletizing equipment to form pellets of 4 mm in diameter and 4 mm in length, as depicted in Figure 1. From each treatment outlined in Table 3, 500 g were obtained to prepare the samples and perform physical property characterization tests.

This extrusion/pelletizing process was parameterized according to works on the extrusion of plastics by Rodriguez, P., Prieto, E., Pachón, Palange, C., Marcus, A., Scurr, D., Stephen, P., Eichhorn, J. [16,19].

Injection Molding of Test Specimens.

**Figure 2:**
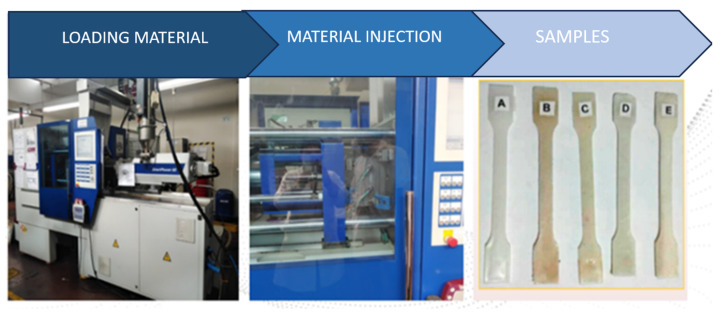
Injection molding process of test specimens. (A) LDPE 100%, PS 0.0%, MFCA 0. 0%; (B) LDPE 84.95%, PS 15%, MFCA 0. 05%; (C) LDPE 84.85%, PS 15%, MFCA 0.15%; (D) LDPE 84.7%, PS 15%, MFCA 0.30%; (E) LDPE 85%, PS 15%, MFCA 0.00%.

For the production of test specimens in accordance with ASTM D-638 [20], used for tensile mechanical tests, the injection molding process was utilized with a two-cavity cold runner and automatic ejection metal mold. The materials developed with each of the established treatments were preconditioned in a SHINI brand pre-dryer set to a temperature of 80 °C for 15 min to remove moisture. They were then loaded into the hopper of the injection machine, where they gravitated into the screw chamber and were homogenized and plasticized before being injected into the mold, as shown in Figure 2.

When the material is injected into the mold, it is cooled by water circulation within the mold tailored to the geometry of the part to ensure rapid solidification. The mold then opens, and the part is ejected by an ejection system and retrieved by a robotic arm that places it on a conveyor belt for final collection.

The injection molding process for the potato starch with low-density polyethylene was programmed according to the parameters detailed in the research work of Rodríguez, P., Prieto, E., Pachón, Y. [16].

### 2.3. Material Characterization

Tensile Strength Resistance

Tensile strength tests were conducted using a BESMAK (Sincan/Ankara, Turquía) brand universal testing machine with a 5-ton capacity at the SENA Center for Materials and Testing in the regional District Capital. Dogbone-shaped specimens following ASTM D 638 were obtained by injection molding from the various treatments (T1, T2, T3, and T4). This tensile test was performed at a speed of 5 mm/s until the breakpoint.

Hardness

Shore D hardness is used, taking into account that its resistance to indentation will be measured, thus indicating its rigidity and wear resistance. This measurement is essential for comparing the rigidity of different polymers, which helps in the selection of the appropriate material for specific applications.

Hardness is measured by the depth of indentation of the indenter, according to ASTM D 2240 [21]. It is one of the mechanical tests that provides very valuable information about the properties related to resistance to penetration and/or scratching of materials. A CHECK-LINE brand durometer, model MSDD 4AD00 for Shore D hardness, was used. This device is located in the laboratory for composite materials and polymers at the SENA Center for Materials and Testing. The test was carried out at different points on the specimens from the treatments (T1, T2, T3, and T4), where a penetration force was applied, leaving an impression on the material, which allowed for the determination of the corresponding hardness value for each treatment.

Melt Flow Index (MFI)

The MFI is a basic rheological test performed on a polymer to determine its flowability. It is measured in grams per 10 min and is defined as the amount of material (measured in grams) that flows through the orifice of a capillary die in 10 min while maintaining constant standard pressure and temperature according to ASTM D 1238 [22]. The test was conducted using an ATLAS brand plastometer, reference MFI-2, located in the polymer laboratory at the ASTIN National Technical Assistance Center to the industry of SENA regional Valle.

Scanning Electron Microscopy (SEM)

To observe and compare the morphology of the types of microfibers corresponding to the parameters set and shown in Table 1, the scanning electron microscopy (SEM) technique was employed. Micrographs were directly observed in the fracture area of the specimens of each material with treatments T1, T2, T3, and T4 to monitor the behavior of the fibers. Samples were mounted with copper tape to enhance conductivity. A PHENOM XL brand scanning electron microscope was used, which is located at the SENA center for composite materials and polymers and Testing Laboratory.

## 3. Results

The results obtained in the present research will be described below.

### 3.1. Electrospinning Process

**Figure 3:**
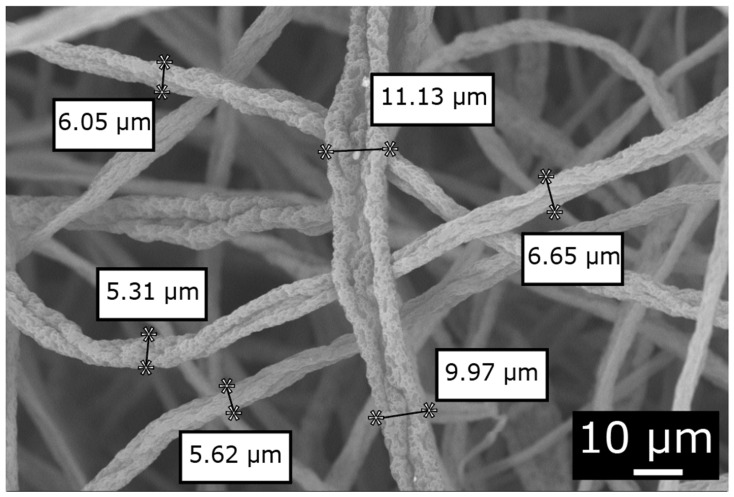
Display of the fiber diameter by SEM of sample T1 2.5 kX.

In the electrospinning process, trials were conducted with the previously established variables, which achieved an average fiber diameter of 7.46 ± 2.5 µm, with fibers as thin as 5.31 µm and as robust as 11.13 µm. A surface roughness on each of the fibers and an excellent structure were also observed, as shown in Figure 3.

### 3.2. Characterization of Developed Materials

Tension Tests

Table 4 presents the tensile test results for the different treatments, where it is evident that the treatments reinforced with MFCA (T1, T2, and T3) exceed the ultimate strength value with values over 11.790 MPa, compared to treatment T4, which does not have reinforcement with cellulose acetate microfibers and shows a value of 11.02 MPa.

An analysis was also conducted in comparison to the research studies on composite materials with 15% starch carried out by the SENA Materials and Testing Center—GIMES and the Pedagogical and Technological University of Colombia UPTC—DANUM [15,16], where a similar tensile strength behavior is demonstrated. The T1 sample (LDPE 84.95%, PS 15%, MFCA 0.05%) has an average ultimate strength value of 12.16 ± 0.8 MPa, which is a 10.34% increase relative to treatment T4. The T2 treatment (LDPE 84.85%, PS 15%, MFCA 0.15%) has an average ultimate strength value of 11.79 ± 0.3 MPa, which is a 6.98% increase relative to T4, and the T3 treatment (LDPE 84.7%, PS 15%, MFCA 0.30%) has an average ultimate strength value of 12.32 ± 0.5 MPa, an 11.79% increase over T4.

**Figure 4:**
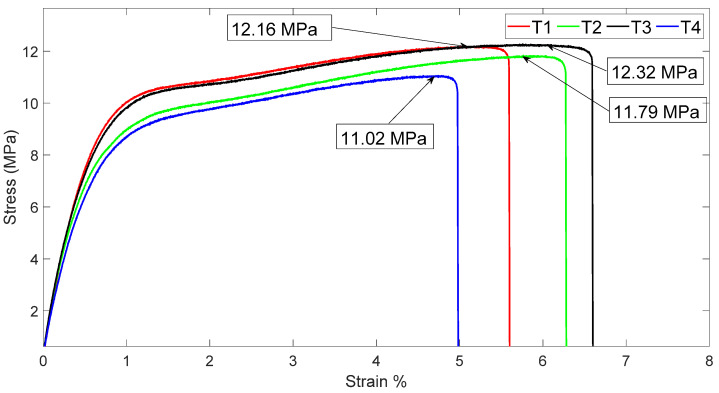
Tensile strength of the treatments made with low-density polyethylene and potato starch.

These results suggest that a higher percentage of cellulose microfibers increases the ultimate tensile strength, which could be explained by their high aspect ratio and high tensile strength. These microfibers can provide greater cohesion and strength to the material, resulting in increased tensile resistance, as evidenced in Figure 4.

Hardness Test

For hardness results, the T4 treatment without MFCA reinforcement showed a value of 54.95 Shore D. For the T1 treatment, the average value was 56.25 Shore D, indicating an increase in hardness of 2.36% compared to T4. The T2 treatment (LDPE 84.85%, PS 15%, MFCA 0.15%) had an average hardness value of 54.60 Shore D, a decrease of 0.63% compared to T4. The T3 treatment (LDPE 84.7%, PS 15%, MFCA 0.30%) had an average hardness value of 52.10 Shore D, decreasing by 5.18% compared to T4.

**Figure 5:**
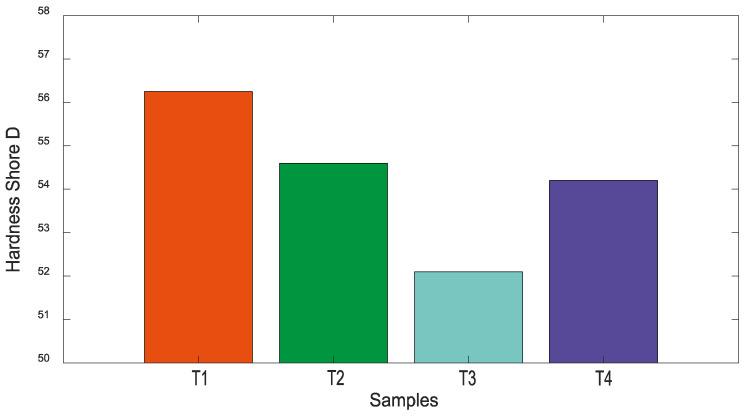
Hardness values of treatments made with potato starch and cellulose microfibers.

The results, which will be shown in Figure 5, suggest that hardness decreases as the amount of microfibers increases within the developed materials; however, it is noted that the T1 sample has a hardness 1.3 Shore D higher than the T4 sample, with only 0.15% MFCA content.

The findings from the research of authors T. Khan, M. T. B. Sultan, and A. H. Ariffin indicate that a higher percentage of reinforcement increases hardness. However, the hardness values obtained in the current study reveal that only one treatment (T1) shows an increase in hardness compared to T4; yet as more microfiber content is added, the hardness begins to decrease, even falling below the unreinforced T4 treatment [5].

Upon examining the hardness values, it can be evidenced that the T2 treatment has a hardness of 54.6, only 0.35 Shore D lower than the T4 treatment, suggesting that these two treatments (T2 and T4) have similar hardness behavior.

Melt Flow Index (MFI)

This test was conducted by measuring the melt flow index of four cuts per treatment and calculating the average value for each sample as recorded in Table 5.

The melt flow index for the material used in the test, low-density polyethylene from ECOPETROL, reference POLIFÉN 641, has a value of 1.80 to 2.10 g/10 min. The T4 treatment of this reference had a melt flow index value in pellet form of 1.90 g/10 min.

The average value for the T1 treatment was 1.69 g/10 min and for the T3 treatment was 1.55 g/10 min. These two treatments are below the limit of the T4 treatment, which is 1.95 g/10 min, with T3 being the treatment with the lowest melt flow index.

On the other hand, the T2 treatment had a melt flow index of 1.91 g/10 min, which is within the limits of the technical data sheet for the reference polyethylene (1.80 to 2.10 g/10 min) and very close to the value of T4.

Scanning Electron Microscopy (SEM)

**Figure 6:**
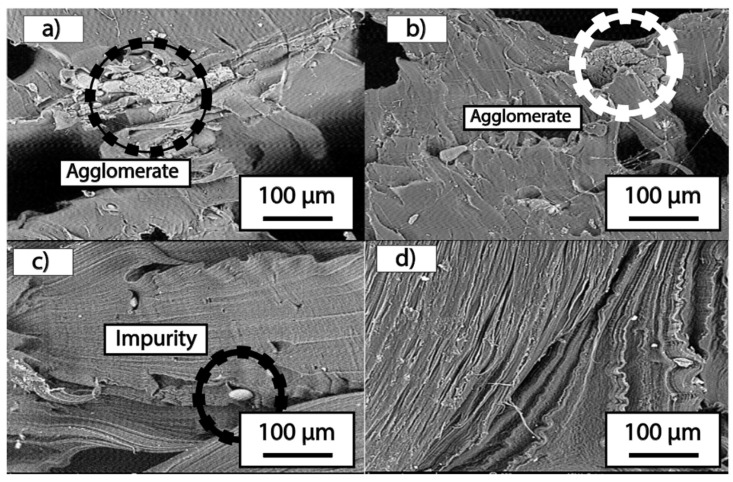
SEM Images of treatments 500× (**a**) T1, (**b**) T2, (**c**) T3, (**d**) T4.

The micrographs were taken from the section of the fractured specimen following the tensile tests of the four developed treatments. The failure area was observed under the scanning electron microscope, from which images were captured and are presented in Figure 6.

In Figure 6a,b, we can observe that in the T1 and T2 treatments, the MFCA creates bonds between the potato starch and the LDPE, leading to agglomerations. However, the MFCA fibers are not detailed, which is attributed to the extrusion process of the three materials that constitute the material for each of the treatments.

Comparing the T1 and T2 treatments with treatment T3, corresponding to image c, it can be seen that the material presents better homogeneity as no agglomerations are observed in its structure. This is due to the bonds created by the MFCA, which contribute rigidity to the material.

In treatment T4, shown in Figure 6d, a structural orientation is observed, which is due to the stretching applied during the tensile test. Since this T4 treatment does not include any MFCA fiber reinforcement, no agglomerations are visible.

In Figure 6c, the presence of impurities within the polymeric matrix is evident; however, there are no agglomerations as observed in Figure 6a,b, indicated by the black and white circles, respectively.

## 4. Discussion

Through the electrospinning process and characterization of the microfibers, it was possible to obtain cellulose acetate microfibers (MFCA) with an average diameter of 7.46 ± 2.5 µm, with a range from 5.31 µm to 11.13 µm. The surface roughness observed in these microfibers can contribute to a better adhesion within the polymeric matrix. This characteristic is crucial since a good interface between the fibers and the matrix is essential to improve the mechanical properties of the composite material. In the study carried out by Syed et al. [23] they demonstrated that the electrospinning of cellulose acetate nanofiber reinforced with activated carbon in a mixture of acetone and DMF solvent 2:1 took 3 h to finish with a centrifugation speed of 0.2 mL/h using a voltage of 15 kV. The surface-modified electrospun fiber membrane adheres to pseudo-first-order kinetics and is collected on a flat aluminum foil placed 8 cm away from the needle tip with a removal efficiency of 98.5%.

Tensile tests revealed that the MFCA-reinforced composites (T1, T2, and T3 treatments) showed a significant improvement in ultimate stress compared to the T4 treatment (no reinforcement). Specifically, the T3 treatment (0.30% MFCA) achieved an ultimate stress of 12.32 ± 0.5 MPa, representing an increase of 11.79% over the T4 treatment. This confirms that MFCA reinforcement, due to its high length-to-diameter ratio and tensile strength, improves the mechanical strength of the composite. In the study conducted by Saraswat P, Singh B [24], it shows that 1:3.5 is the optimized plastic to aggregate ratio for LDPE-RCA composite paver blocks with compressive strength of 20 to 35 MPa, suitable for pedestrian or low traffic areas. Indicating that it is crucial to achieve better interfacial bonding, particle stress transfer and ductile behavior, leading to substantial energy absorption before failure.

SEM micrographs provided a detailed view of the internal structure of the composites. Images of treatments T1 and T2 showed agglomerations of MFCA, indicating non-optimal dispersion. However, T3 showed a more homogeneous structure with no visible agglomerations, which could explain the improvement in tensile strength observed in this treatment. The image of treatment T4 confirmed the absence of reinforcement, showing an oriented and stretched structure due to the lack of reinforcing fibers. In the study conducted by Saraswat P, Singh B [24], they show scanning electron micrographs where they reveal the intricate interfacial transition zones (ITZ) within the composite. The well-sorted aggregates, uniform dispersion of LDPE, and robust ITZ formation between LDPE and RCA contribute to better stress transfer, improved orientation, and reduced porosity. Proper interfacial bonding is essential for the mechanical strength and overall durability of the material.

## 5. Conclusions

The process of electrospinning for the acquisition of cellulose acetate microfibers yielded a fiber diameter standard deviation of 7.46 ± 2.5 µm with a rough surface, successfully standardizing the process for continuous production as per the experimental design proposed for each treatment.

In the mechanical tensile tests, the T3 treatment (LDPE 84.7%, PS 15%, MFCA 0.30%) showed the best result; however, the other two treatments with microfiber reinforcement increase this ultimate strength property of a polymer by 15% compared to a synthetic thermoplastic polymer.

In the hardness tests, the hardness of the T3 treatment (LDPE 84.7%, PS 15%, MFCA 0.30%) is lower than that of a synthetic material, in this case, low-density polyethylene, indicating it is a material that does not easily allow penetration.

In the melt flow index tests, treatments T1 and T3 show a reduction in melt flow index compared to treatment T4, reaching values below 1.7 (g/10 min), while for treatment T2, a melt flow index of 1.91 ± 0.13 (g/10 min) was achieved, close to the T4 treatment of 1.95 ± 0.09 (g/10 min). This indicates that the most suitable treatment for the extrusion process is T2, as it falls within the recommended melt flow index range for LDPE according to its technical data sheet.

The reinforcement with cellulose acetate microfibers improves the mechanical properties of the T4 material, which is solely composed of LDPE and PS. It was observed that a load below 0.3% significantly affects its mechanical properties, referring to various studies used as benchmarks in this research.

This composite material with a thermoplastic matrix reinforced with PS and microfibers (MFCA) makes it a potential candidate for use in industrial processes of this kind for the manufacture of products, providing an alternative to synthetic polymers.

## Figures and Tables

**Table 1:** Standardization of electrospinning process parameters.

Parameters	Values
Solution flow rate	30 mL/hra
Voltage	17 kV
Distance between needle and collector	30 cm
Volume	5 mL
Polymer concentration	6%

**Table 2:** Experimental Design Treatments.

Treatments			
T	Mixtures (%)		
	LDPE	PS	MFCA
T_1_	84.95	15	0.05
T_2_	84.85	15	0.15
T_3_	84.7	15	0.30
T_4_	85	15	0.00

**Table 3:** Extrusion/pelletizing parameter sheet.

Process Parameters: Extrusion/Pelletizing	
Temperatures	Zone 1: 50 °C
	Zone 2: 80 °C
	Zone 3: 100 °C
	Zone 4: 140 °C
	Zone 5: 165 °C
Cooling Tank Temperature	10 °C
Extruder Screw Speed	30 RPM
Pelletizer Speed	40 RPM
Extruder Motor Frequency	50 Hz
Pelletizer Motor Frequency	60 Hz

**Table 4:** Experimental design treatments.

Treatment	Ultimate Strength
T1 (LDPE 84.95%, PS 15%, MFCA 0.05%)	12.16 ± 0.8 MPa
T2 (LDPE 84.85%, PS 15%, MFCA 0.15%)	11.79 ± 0.3 MPa
T3 (LDPE 84.7%, PS 15%, MFCA 0.30%)	12.32 ± 0.5 MPa
T4 (LDPE 85%, PS 15%, MFCA 0.00%)	11.02 ± 0.2 MPa

**Table 5:** Melt flow index values for each treatment.

Treatment Identification	Average Melt Flow Index (g/10 min)
T1	1.69 ± 0.01
T2	1.91 ± 0.13
T3	1.55 ± 0.08
T4	1.95 ± 0.09

## Data Availability

The original contributions presented in the study are included in the article, further inquiries can be directed to the corresponding author.
